# *PRTFDC1* Is a Genetic Modifier of HPRT-Deficiency in the Mouse

**DOI:** 10.1371/journal.pone.0022381

**Published:** 2011-07-27

**Authors:** Alaine C. Keebaugh, Heather A. Mitchell, Meriem Gaval-Cruz, Kimberly G. Freeman, Gaylen L. Edwards, David Weinshenker, James W. Thomas

**Affiliations:** 1 Yerkes National Primate Research Center, Atlanta, Georgia, United States of America; 2 Department of Human Genetics, Emory University School of Medicine, Atlanta, Georgia, United States of America; 3 Department of Physiology and Pharmacology, University of Georgia, Atlanta, Georgia, United States of America; Institut Jacques Monod, France

## Abstract

Lesch-Nyhan disease (LND) is a severe X-linked neurological disorder caused by a deficiency of hypoxanthine phosphoribosyltransferase (HPRT). In contrast, HPRT-deficiency in the mouse does not result in the profound phenotypes such as self-injurious behavior observed in humans, and the genetic basis for this phenotypic disparity between HPRT-deficient humans and mice is unknown. To test the hypothesis that HPRT deficiency is modified by the presence/absence of phosphoribosyltransferase domain containing 1 (*PRTFDC1*), a paralog of HPRT that is a functional gene in humans but an inactivated pseudogene in mice, we created transgenic mice that express human *PRTFDC1* in wild-type and HPRT-deficient backgrounds. Male mice expressing *PRTFDC1* on either genetic background were viable and fertile. However, the presence of *PRTFDC1* in the HPRT-deficient, but not wild-type mice, increased aggression as well as sensitivity to a specific amphetamine-induced stereotypy, both of which are reminiscent of the increased aggressive and self-injurious behavior exhibited by patients with LND. These results demonstrate that *PRTFDC1* is a genetic modifier of HPRT-deficiency in the mouse and could therefore have important implications for unraveling the molecular etiology of LND.

## Introduction

The first mouse genetic model of a human disease developed by germline transmission of a selected mutation in embryonic stem cells was Lesch-Nyhan disease (LND, MIM: 300322) [1], [2], [3]. LND is an X-linked metabolic disease is caused by mutations in a purine salvage enzyme, hypoxanthine phosphoribosyltransferase (HPRT, EC 2.4.2.8), and results in profound developmental, neurological and behavioral phenotypes, including aggressive and self-injurious behavior [4]. While HPRT-deficiency in the mouse does result in reduced basal ganglia dopamine (DA) [5], [6], which is thought to be important for the neurobehavioral phenotypes observed in LND [7], except for an increased sensitivity to amphetamine, no other behavioral phenotypes have been associated with the loss of HPRT activity in the mouse [6], [8]. The phenotypic disparity between humans and mice deficient for HPRT led to the hypothesis that purine metabolism differed in some way between these two species, and that uncovering of the basis for such a difference would likely provide important insights into LND [6], [9]. However, attempts to improve the mouse model of LND by generating double mutants of HPRT and other purine metabolic enzymes have failed to identify genetic factors that contribute to the interspecies phenotypic disparity in HPRT-deficiency [4], [10].

Genetic alteration of the mouse genome to account for differences in gene content between humans and mice can be critical for creating more accurate models of human disease, e.g. [11], [12], [13]. Previously, we reconstructed the evolutionary history of the *HPRT* gene family in vertebrates and inferred that a paralog of *HPRT*, called phosphoribosyltransferase domain containing 1 (*PRTFDC1*), arose via a duplication of *HPRT* prior to the radiation of vertebrates [14]. Despite its antiquity and presence in the genome of humans and all other sampled vertebrates, we discovered that *PRTFDC1* had been inactivated in the mouse lineage [14]. Two previous observations lead us to hypothesize that the presence of *PRTFDC1* in humans and the absence of a functional copy of this gene in mice could contribute to the phenotypic disparity of HPRT-deficiency between those species. First, since there is unvalidated evidence that the HPRT and PRTFDC1 proteins may physically interact with one another [15], the regulation, or activity of PRTFDC1 might be altered when HPRT is absent or mutated. Second, though it is not known if PRTFDC1 has biologically relevant phosphoribosyltransferase activity [16], because the activity of other phosphoribosyltransferases with known enzymatic activity has been reported to be increase in the absence of HPRT due to elevated levels of a common substrate, 5-phosphoribosyl-1-pyrophosphate (PRPP) , reviewed in [4], the activity of PRTFDC1 has the potential to be similarly affected. We therefore proposed that when HPRT is absent or mutated, the altered activity and/or protein-protein interactions of PRTFDC1 enhance the phenotypic severity of HPRT-deficiency. Thus, the lack of a functional *PRTFDC1* gene in the mouse effectively suppresses the HPRT-deficient phenotypes in that species. Here we report the results of our experiments designed to empirically test for a genetic interaction between *HPRT* and *PRTFDC1* in the mouse.

## Materials and Methods

### Generation and breeding of the transgenic mice

All experimental protocols using mice were approved by the Emory University IACUC and meet the guidelines of the American Association for Accreditation of Laboratory Animal Care. The IACUC approval number for this study is 192-2008. Human bacterial artificial chromosome (BAC) clone RP11-129O7 was used to generate transgenic mice by pronuclear injection into an FVB background. Four transgenic founders were identified by PCR using primers specific for the human BAC (see Materials and Methods S1). The transgenic lines were expanded and maintained as heterozygotes while being backcrossed into C57BL/6 (Charles River strain 027). Mice carrying a null allele of *Hprt* [2] that had been bred into the C57BL/6 background were purchased from The Jackson Laboratory (B6.129P2-*Hprt1^b-m3^*/J, stock number 002171) and maintained as a closed breeding colony. After five generations of backcrossing into the C57BL/6 background, males hemizygous for *Tg(RP11-129O7)9* and *Tg(RP11-129O7*)*13* were crossed to females (*Hprt* ^−/−^, *Hprt* ^+/−^ and *Hprt* ^+/+^) from the *Hprt* colony to produce wild-type and HPRT-deficient males with or without the transgene. Protocols used to genotype the transgenic and HPRT-deficient mice are provided in the Materials and Methods S1.

### Behavioral assays

#### General

All mice were reared in a specific pathogen-free facility with a 12 hr light/dark cycle (lights on - 7 am; lights off - 7 pm). Food and water were available ad libitum except during behavioral testing. All experiments were carried out in a quiet, isolated behavior room between 8:00 am and 5:00 pm. Mice were moved to this room at least 24 hr before testing.

#### Locomotor activity

Locomotor activity was assessed using an automated system (San Diego Instruments, La Jolla, CA, USA) with photobeams that recorded ambulations (consecutive beam breaks). For exploratory behavior, mice were placed individually in the chambers and allowed to explore for 2 hr, and all ambulations recorded. For circadian activity, mice were kept in the chambers following their novel activity test for an additional 24 hr. Total ambulations were recorded.

#### Amphetamine-induced stereotypy

Amphetamine-induced stereotypy was assessed as we have described previously [17]. Drug naïve mice were placed individually in locomotor-monitoring chambers for 2 hr and then injected with amphetamine (5 mg/kg, i.p.). 30 min after injection, mice were videotaped for a 30-min test. The test was scored for stereotypy by a trained and blinded observer by breaking the 30 min into 10-sec bins, and recording the predominant behavior the mouse was engaged in during each bin.

#### Resident-Intruder aggression

Resident-intruder aggression was assessed as we have described previously [18]. An intruder male mouse was introduced into the cage of the resident mouse for a 5-min videotaped session. Events were scored by a trained observer blind to genotype and included whether the resident mouse attacked the intruder mouse, latency to first attack, and number of attacks. This test was then repeated twice, with two days in between each test, for a total of three trials.

### Statistical analysis

All data is presented as mean ± SEM. Exploratory locomotor behavior and nail-biting stereotypy were analyzed using a one-way ANOVA. Circadian locomotor activity, aggression latency, and aggression attack number were analyzed with a two-way repeated measures ANOVA. Stereotypy behavior was analyzed with a one-way ANOVA. Bonferroni post hoc tests were conducted following ANOVA analysis. Percentage of attackers was assessed with a Chi square contingency table, followed by Bonferroni post hoc tests, adjusted for multiple comparisons. Statistical analysis was conducted using Graphpad™ Prism 4.0c for Macintosh or PC (San Diego, CA).

### Monoamine neurotransmitter assays

Mice were sacrificed by CO_2_, decapitated, the brains removed, and the regions of interest dissected on ice. HPLC analysis with electrochemical detection was performed as described previously [19] with minor modifications.

### Tissue collection and gene and protein expression assays

Tissues were dissected from p0, p28 and 7 week-old males, immediately frozen on dry ice and stored at −80°C. Total RNA was isolated from the tissues with either the RNeasy Lipid Tissue Mini Kit (Qiagen) or Trizol (Invitrogen). The isolated mouse total RNA, and total human RNA (Clontech and US Biologics) was used as a template to generate cDNA (in duplicate) using Superscript II Reverse Transcriptase (Invitrogen) primed with an oligo(dT) primer according to manufacturer's recommendations. Quantitative RT-PCR reactions (n = 4 replicates per cDNA template) were performed using Platinum® SYBR® Green qPCR SuperMix-UDG (Invitrogen) and Roche LightCycler V3 using commercially available primers for human *PRTFDC1*, and species-specific primers for *HPRT1*, *POLR2A*, and *ACTB* (SuperArray Bioscience Corp.). Western blots were performed using protein extracts from mouse tissue probed with antibodies to PRTFDC1 (ProteinTech Group, Inc, 11986-1-AP) and a control protein, ACTB (Santa Cruz Biotechnology, Inc, sc-20975).

## Results

### Mice expressing *PRTFDC1* are viable and fertile

Four lines of transgenic mice (*Tg(RP11-129O7)9*, *13*, *14* and *36*) were generated using a human BAC clone that contains the *PRTFDC1* locus (Fig. S1). RT-PCR confirmed that the human *PRTFDC1* gene was expressed in all the transgenic lines (Fig. S2). In the wild-type genetic background males and females hemizygous for the transgene from all the transgenic lines were viable, fertile, and visibly indistinguishable from their littermates. Two of the transgenic lines, *Tg(RP11-129O7)9* and *Tg(RP11-129O7)13*, were arbitrarily selected to create HPRT-deficient mice expressing the transgene (hereafter designated as *Hprt^−/0^*/Tg) and subjected to more detailed molecular and phenotypic characterization. The *Hprt^−/0^*/Tg males were viable, fertile, and did not differ with respect to their wild-type, HPRT-deficient (*Hprt^−/0^*), or PRTFDC1 trangenic (Tg) littermates in any gross phenotypes. The presence of the transgene also had no effect in either the wild-type or *Hprt^−/0^* background on exploratory or circadian locomotor activity (Fig. S3A and B).

### Increased aggression in HPRT-deficient mice expressing *PRTFDC1*

**Figure 1:**
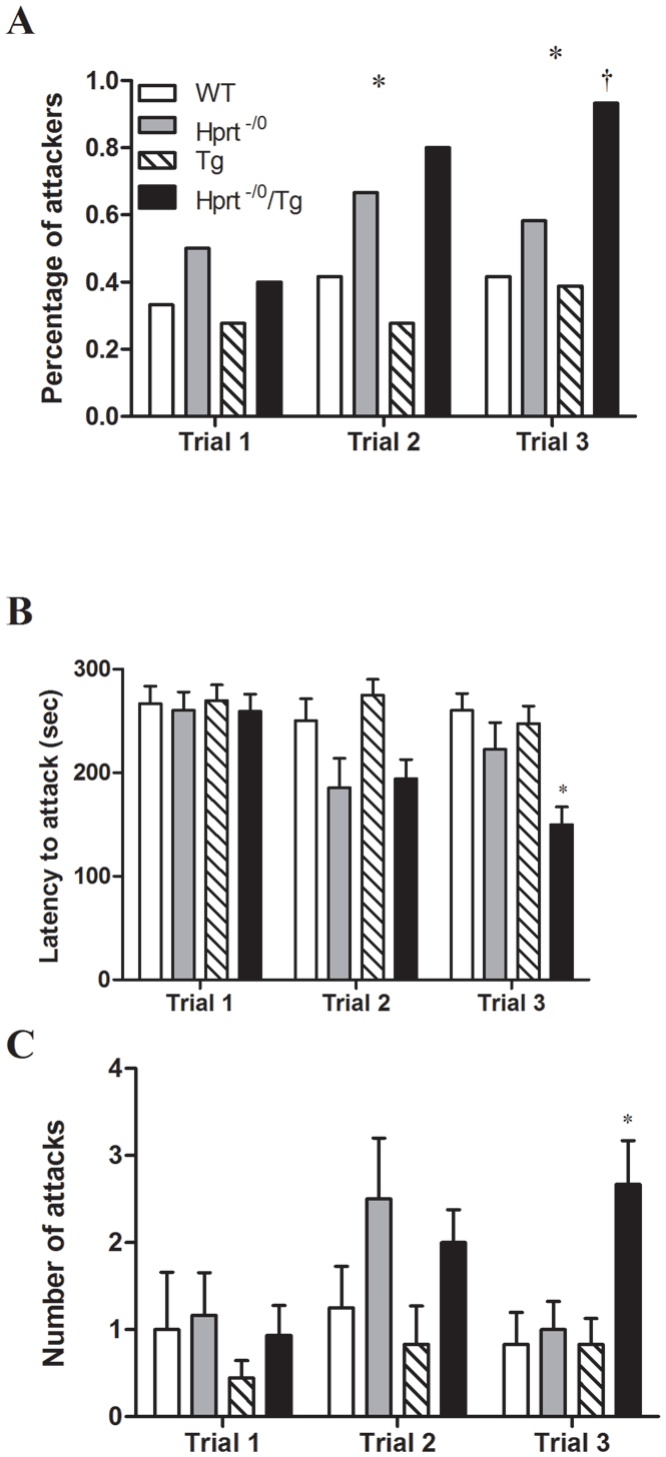
Resident-Intruder aggression. Mice (n = 11–17 per genotype) were tested for intruder aggression in three separate trials, with two days between trials. (A) Percentage of residents that attacked the intruder at each trial. * P<0.05 for overall differences between genotypes within the same trial trial. † P<0.05 comparing *Hprt^−/0^*/Tg to wild-type (WT) and Tg within the same trial. (B) Mean latency to attack ± SEM for each genotype during each trial; * P<0.05 comparing *Hprt^−/0^*/Tg to all other genotypes within the same trial. (C) Number of attacks ± SEM for each genotype during each trial; * P<0.05 comparing *Hprt^−/0^*/Tg to all other genotypes within the same trial.

LND patients tend to display an increased level of aggressive behavior [20]. To determine if the expression of *PRTFDC1* increased aggressive behavior in the HPRT-deficient mice, we performed a resident-intruder test on wild-type, *Hprt^−/0^*, Tg, and *Hprt^−/0^*/Tg males. The aggression of the *Hprt^−/0^*/Tg males increased in each successive trial, whereas a similar trend of increasing aggression was not observed in the other genotypes (Fig. 1A–C). As a consequence, in the third trial the *Hprt^−/0^*/Tg males had the highest percentage of individuals that attacked the intruder (Fig. 1A), displayed significantly shorter latency to attack (Fig. 1B), and significantly higher number of attacks (Fig. 1C) than the other genotypes. A two-way repeated measures ANOVA of latency to first attack revealed a significant interaction effect between genotype and trial (F(6,106) = 3.981, p = 0.0012), as well as main effects of both genotype (F(3,106) = 4.533, p = 0.0067) and trial (F(2,106) = 10.66, p<0.001). When number of attacks were analyzed, there was a main effect of both genotype (F(3,106) = 2.942, p = 0.0413) and trial (F(2,106) = 4.459, p = 0.0138), in addition to a significant interaction between genotype and trial (F(6,106) = 2.375, p = 0.0342). While the *Hprt^−/0^*males did display the highest average number of attacks in the second trial this result was primarily due to a single individual and not was not significantly different than the other genotypes.

### Increased sensitivity to amphetamine-induced stereotypy in HPRT-deficient mice expressing *PRTFDC1*

**Figure 2:**
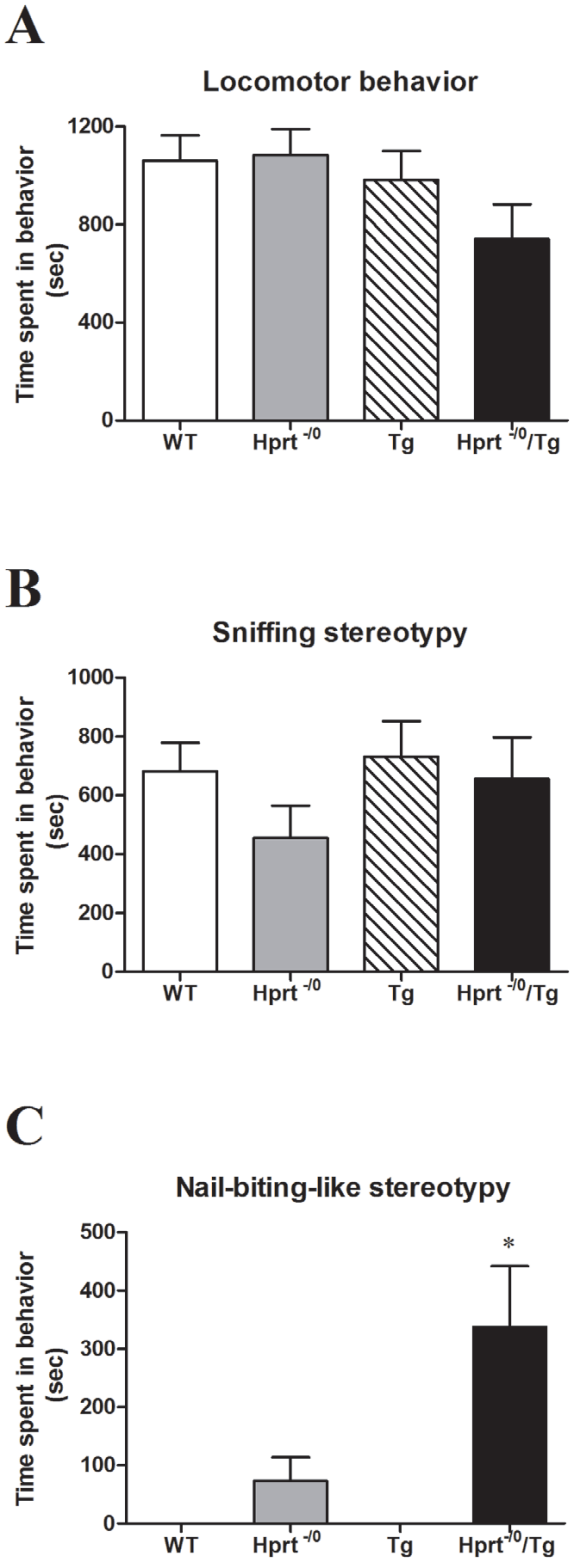
Amphetamine-induced stereotypy. Mice (n = 11–17 per genotype) were scored for stereotypy behaviors following the administration of 5 mg/kg amphetamine. Predominant behaviors were locomotor behavior (A), sniffing stereotypy (B), and nail-biting-like stereotypy (C). Shown is mean time of each behavior ± SEM during the 30 min recorded test. * P<0.05 comparing *Hprt^−/0^*/Tg to all other genotypes. No differences were seen between genotypes for locomotor behavior or sniffing stereotypy.

Due to the DA deficit in the basal ganglia, it was hypothesized these *Hprt^−/0^* mice might be more sensitive to drugs that interact with the DA systems and it was reported that mice deficient for HPRT can be more sensitive to amphetamine-induced stereotypic behavior [8]. To assess if the presence of the transgene affected the behavioral response of *Hprt^−/0^* males to amphetamine, we quantified and compared the behavior of wild-type, *Hprt^−/0^*, Tg, and *Hprt^−/0^*/Tg males after the administration of amphetamine (5 mg/kg, i.p.). Amphetamine induced two main types of behaviors in all genotypes: locomotor behavior and excessive sniffing stereotypy. These behaviors were seen at comparable levels in all genotypes, though there is a trend towards decreased locomotion in the *Hprt^−/0^*males (Fig. 2A–B). However, the *Hprt^−/0^*/Tg mice did significantly differ from all other genotypes with respect to a peculiar stereotypical behavior that was only observed following the administration of amphetamine (Fig. 2C). The majority (9/15) of *Hprt^−/0^*/Tg displayed a distinctive hunched posture, with their forepaws off the ground, and their head angled downwards. The mice brought their forepaws toward their mouths in a repetitive motion, while bobbing their heads toward the paws, and appeared to be biting or chewing (Video S1). This behavior is henceforth referred to as nail biting, although subsequent examination of the paws and nails did not reveal any physical damage. Shorter bouts of his behavior were observed in 6 out of the 11 *Hprt^−/0^* males, but never in the Tg (n = 18) or wild-type (n = 12) animals. Other stereotypy behaviors including freezing, head bobbing, and rearing were also seen, though to a very minimal extent, in mice of all genotypes.

### *PRTFDC1* expression does not alter neurotransmitter levels in the brain

**Figure 3:**
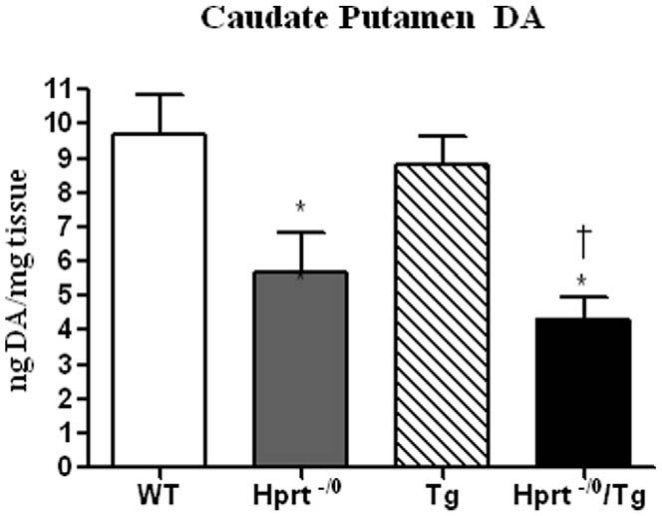
Dopamine levels in the caudoputamen. DA levels were measured in wild-type (n = 9), *Hprt^−/0^* (n = 7), Tg (n = 12) and *Hprt^−/0^*/Tg (n = 14) males. Data shown is ng/mg of tissue ± SEM. * P<0.05 comparing *Hprt^−/0^* and *Hprt^−/0^*/Tg to wild-type. † P<0.05 comparing *Hprt^−/0^*/Tg to wild-type and Tg.

A reduction in DA levels in the basal ganglia is thought to be important for the neurobehavioral phenotypes observed in LND [7]. HPRT-deficiency in the mouse also results in a reduction in dopamine levels in the basal ganglia, albeit to a lesser degree than is observed in LND patients [5]. To determine if the presence of the transgene altered the level of DA (or other monoamine neurotransmitters) in wild-type and *Hprt^−/0^* genetic backgrounds, we compared the levels DA, norepinephrine (NE), epinephrine, DOPAC, 5-HIAA, 5-HT, and HVA in the caudate putamen (CP), nucleus accumbens (NAc), and olfactory bulb (OB) across genotypes. Consistent with previous reports, DA levels were significantly reduced in the *Hprt^−/0^* (n = 7) compared to the wild-type mice (n = 9) in both sampled regions of the basal ganglia, the CP and NAc (p<0.05, see Fig. 3 and Fig. S4A), but not the OB (Fig. S4B). The presence of the transgene did not result in a significant difference in the DA levels in either the wild-type or *Hprt^−/0^* genetic backgrounds (Fig. 3 and Fig. S4), though there was a trend toward lower DA levels in the CP of *Hprt^−/0^/*Tg compared to *Hprt^−/0^* males (Fig. 3). Thus, the behavioral phenotypes we observed in the *Hprt^−/0^*/Tg males were not associated with a further reduction of DA in the basal ganglia. No significant differences in the level of norepinephrine, epinephrine, DOPAC, 5-HIAA and 5-HT were observed between genotypes in any of the sampled brain regions.

### *PRTFDC1* is expressed in tissues affected in LND

**Figure 4:**
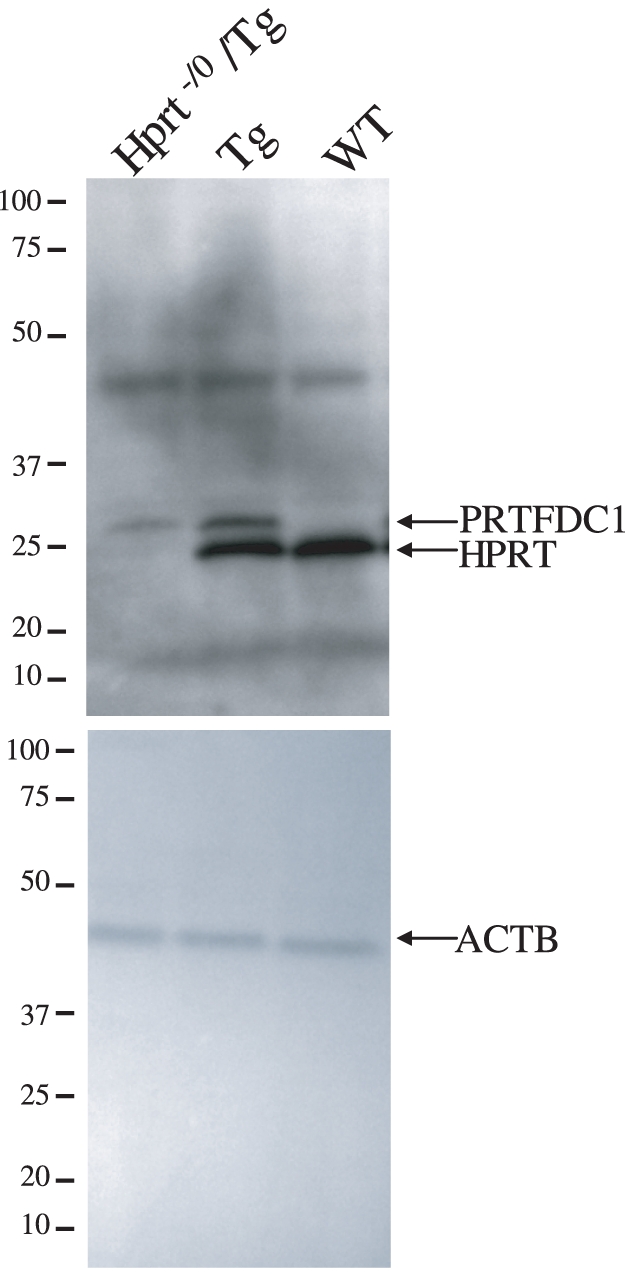
PRTFDC1 protein expression in the transgenic mice. Western blots of 7 week old male whole brain protein extracts from *Hprt^−/0^*/Tg, Tg, and wild-type mice from transgenic line 9 were probed with an antibody to PRTFDC1, which has a molecular weight of 25.7 kDa (top panel). Note the PRTFDC1 antibody cross-reacts with HPRT (molecular weight = 24.6 kDa) and that the two proteins are 68% identical and 83% similar. A β-actin antibody was used as a loading control (bottom panel). The positions of the molecular weight (kDa) markers are indicated on the left and the proteins are labeled on the right. Also note the band between 37 and 50 kDa in the top panel is leftover signal from probing with the β-actin antibody, and no additional band consistent with HPRT or PRTFDC1 dimers or tetramers were visible on this blot.

The brain and testes are two tissues directly affected in male LND patients [4] and database searches indicated that *PRTFDC1* is expressed in those and other tissues in normal humans. To empirically determine if *PRTFDC1* is expressed in normal human brain and testes, and if that were also the case in the transgenic mice, we quantified the transcript level of *PRTFDC1* and *HPRT* by RT-PCR. *PRTFDC1* and *HPRT* transcripts were detected in the brain, testes and liver at all stages of development assayed in humans (Fig. S5). Likewise, the *PRTFDC1* transgene was expressed in those three tissues at all time-points assayed in Tg and *Hprt^−/0^*/Tg males (Fig. S6), and the PRTFDC1 protein was also detected in Tg and *Hprt^−/0^*/Tg males (Fig. 4).

## Discussion

The molecular connection between deficiency of the purine salvage enzyme HPRT and the developmental and neurobehavioral phenotypes observed in LND patients is unknown [4]. Here we have demonstrated that *PRTFDC1*, a gene that arose from an ancient duplication event of *HPRT*, and is thus linked to purine metabolism by its evolutionary history, is a genetic modifier of HPRT-deficiency in the mouse. Specifically, we did not detect any significant differences between PRTFDC1 transgenic and wild-type mice. However, *Hprt^−/0^*/Tg mice did differ from wild-type, Tg, and *Hprt^−/0^* mice in two phenotypes of relevance to LND; aggression and sensitivity to a specific amphetamine-induced stereotypy. While the phenotypes of the *Hprt^−/0^*/Tg mice do not recapitulate the full spectrum of severe phenotypes observed in LND patients, these results do support a genetic interaction in mice between *HPRT* and *PRTFDC1* in which the presence/absence of *PRTFDC1* acts as an enhancer/suppressor of HPRT-deficiency. Nevertheless, it is also clear from our findings that the presence of a functional copy of PRTFDC1 in humans is not solely responsible for the phenotypic disparity between LND patients and HPRT-deficient mice. Thus, other as of yet unidentified factors must also contribute to the qualitative and quantitative difference in phenotypes caused by the loss of HPRT in mouse and man.

We propose the identification of a genetic interaction between *HPRT* and *PRTFDC1* in the mouse is significant for the following reasons. First, while the brains of *Hprt^−/0^* mice are not normal, e.g. [5], [21], the increased aggression observed in *Hprt^−/0^*/Tg mice is to our knowledge the only spontaneous behavioral phenotype observed in mice deficient for HPRT. Moreover, because increased aggression is a hallmark feature of LND [20], the *Hprt^−/0^*/Tg mice are an improved model for studying that aspect of the human disease. Second, the enhancement of an amphetamine-induced stereotypy in *Hprt^−/0^*/Tg but not Tg males is consistent with the genetic interaction between *HPRT* and *PRTFDC1* affecting striatal function. Depleted levels of DA in the basal ganglia are found in LND patients, and there is strong support for the reduction of DA at an early age being important for the neurological and behavioral phenotypes associated with LND [7]. The increased sensitivity of *Hprt^−/0^* mice to amphetamine but lack of spontaneous neurological and behavioral phenotypes are thought to be a consequence of the milder depletion of DA in the basal ganglia of HPRT-deficient mice compared to LND patients [6], [8]. Though the average DA level in one region of the basal ganglia, the caudate putamen, was lower in *Hprt^−/0^*/Tg versus *Hprt^−/0^* mice, this difference was not significant. Furthermore, none of the other assayed neurotransmitter levels were altered by the presence of the transgene. Thus, while we cannot pinpoint a specific molecular basis for the further increase in sensitivity to amphetamine in *Hprt^−/0^*/Tg mice, further investigation of other aspects controlling dopamine neurotransmission, such as dopamine release and receptor signaling pathways, is warranted.

Finally, the enhancement of the nail-biting like stereotypy in the in *Hprt^−/0^*/Tg is tantalizingly reminiscent of the spontaneous self-injurious behavior, specifically finger biting, observed in LND patients [4] (Video S1). Amphetamine-induced stereotypy was previously reported to be elevated in HPRT-deficient mice [8]. However, because of methodological differences in the quantification and description of amphetamine-induced between the studies, we can not directly compare the frequency of the specific stereotypy we found to be elevated in *Hprt^−/0^* males expressing *PRTFDC1* to those found in [8]. It is important to emphasize that the nail-biting like stereotypy reported here would not qualify as the self-mutilation that has been observed in a rat model of that aspect of LND that is based upon the drug-induced neonatal loss of dopaminergic neurons [22]. Nonetheless, it is striking that the presence of PRTFDC1 in *Hprt^−/0^* mice modified only this one particular stereotypy, which we have rarely if ever observed in any other wild-type or mutant strains, including those with amphetamine hypersensitivity (e.g. [17].

In conclusion, we have demonstrated a genetic interaction between *HPRT* and *PRTFDC1* in the mouse that is relevant to at least a subset of the phenotypes observed in LND. Though PRTFDC1 does contain a phosphoribosyltransferase domain, it has yet to be determined if this protein has significant enzymatic activity [16], or if it perhaps has evolved a regulatory function dependent on the binding of PRPP similar to other members of this gene family [23], [24]. In addition, while *PRTFDC1* has been identified as a potential tumor suppressor [25], [26], how it functions in this capacity is also unknown. Elucidating the function of *PRTFDC1* could therefore provide a key piece of knowledge as to how humans and mice differ in their direct or indirect regulatory interactions with purine metabolites and enzymes, and ultimately could lead to a better understanding and treatment of LND.

## Supporting Information

Materials and Methods S1 **Genotyping methods and the protocol for confirming expression of the transgene transcript.**
(DOCX)

Figure S1 **The genomic location of human BAC clone RP11-129O7.** The position of human BAC clone RP11-129O7 is shown with respect to its position on chromosome 10 (hg18) and *PRTFDC1*. The positions of PCR primers used in the identification of the transgenic founders and genotyping and transgenic mice are also shown.
(EPS)

Figure S2 **The human** ***PRTFDC1*** **gene is expressed in the transgenic mice.** RT-PCR using species and gene-specific primers verified that the human *PRTFDC1* gene was expressed in the four lines of transgenic mice. An example of the RT-PCR results for *Tg(RP11-129O7)13* and *Tg(RP11-129O7)36* p28 whole brain cDNAs is shown (see Materials and Methods S1 for methodological details). The left gel image is of the RT-PCR product for human *PRTFDC1* (200 bp) and the right gel image is a control showing the RT-PCR product for endogenous mouse *Hprt1* (202 bp). Note that *PRTFDC1* expression was also detected in *Tg(RP11-129O7)9* (see Fig. S6) and *Tg(RP11-129O7)14*.
(EPS)

Figure S3 **Neither deficiency of HPRT nor the presence of the PRTFDC1 transgene affect locomotor behavior.** Mice were placed in locomotor-monitoring chambers and ambulations were recorded for 2 (a) or 24 (b) hours. Shown are mean ± SEM ambulations (consecutive beam breaks).
(TIF)

Figure S4 **Dopamine levels in the nucleus accumbens and olfactory bulbs.** DA levels were measured in males (n = 7–14) from each genotype in the nucleus accumbens (a) and the olfactory bulbs (b). * P<0.05 comparing *Hprt^−/0^* males (with or without the transgene) with wild-type and Tg mice. No significant differences between genotypes were seen in the olfactory bulbs.
(TIF)

Figure S5 **Transcript profiles of** ***HPRT1*** **and** ***PRTFDC1*** **in human tissue.** Relative transcript abundance of *HPRT1* (black) and *PRTFDC1* (white) per 1,000,000 *ACTB* molecules. Total human RNA (Clontech and US Biologics) was used to generate cDNA (in duplicate) using Superscript II Reverse Transcriptase (Invitrogen) according to manufacture's recommendations. Quantitative RT-PCR reactions (n = 4 replicates per cDNA template) were performed using Platinum® SYBR® Green qPCR SuperMix-UDG (Invitrogen) and Roche LightCycler V3 using commercially available primers for human *PRTFDC1* and *HPRT1* (SuperArray Bioscience Corp.).
(EPS)

Figure S6 **Transcript profiles of** ***PRTFDC1*** **in wild-type and Hprt-deficient backgrounds.** Relative transcript abundance of *PRTFDC1* in wild-type (black) and *Hprt^−/0^* (white) backgrounds per 1,000,000 *Actb* molecules. No significant differences in the abundance of *PRTFDC1* were observed between genotypes or transgenic lines 9 and 13. Tissues were dissected from neonate (p0), juvenile (p28) and adult (7 week-old) males. Total RNA was isolated and used as a template to generate cDNA using commercially available primers for human *PRTFDC1*, and mouse-specific primers *Actb* (SuperArray Bioscience Corp.).
(EPS)

Video S1 **Nail-biting-like behavior in the** ***Hprt^−/0^*** **/Tg mice.** The *Hprt^−/0^*/Tg exhibit distinct amphetamine-induced stereotypy that is reminiscent of nail-biting. The mice are in an unusual hunched posture and display head-bobbing behavior while simultaneously bringing their forepaws to their mouths and moving their mouths in a chewing or biting manner.
(AVI)
