# Supplementary material for: PRTFDC1 Is a Genetic Modifier of HPRT-Deficiency in the Mouse
Source: PLoS One. 2011 Jul 27;6(7):e22381. doi: 10.1371/journal.pone.0022381 (PMC3144895; doi:10.1371/journal.pone.0022381)
Supplement: Materials and Methods S1 — Genotyping methods and the protocol for confirming expression of the transgene transcript. (DOCX) [file pone.0022381.s001.docx]

**SUPPLEMENTARY MATERIALS AND METHODS**

*Human BAC clone PCR assays*

PCR primers were designed to amplify three positions within the genomic insert of RP11-129O7 and a single location in the BAC vector. The relative position of the primers within the insert is illustrated in Supplementary Fig. 1. PCR reactions for each pair of primers were performed in a 25 μl reaction volume including ~50 ng of genomic DNA, 1.5 units of Taq DNA polymerase (Invitrogen), the manufacturers buffer (-MgCl_2_), 0.8 μM of each primer, 0.2 mM dNTPs, and 1.5 mM MgCl_2_. Cycle conditions were 94°C for 5 minutes, 35 cycles of 94°C for 30s, 58°C for 30s, and 72°C for 1 minute, and a final 7-minute extension at 72°C. The sequence of the PCR primers is as follows:

5’ forward primer: 5'-TCTGTGTGCAGTGTTACCTGTG-3'

5’ reverse primer: 5'-CAAACCAACTGAAAGAGGTTCC-3’

Intron1 forward primer: 5'-TCTCCTGGGATCAGAGTAGACC-3'

Intron1 reverse primer: 5'-ACATCAAGTCCTAAGCGAGAGG-3'

Intron7 forward primer:5'-CTGAAGTCTGCCTATGCAAGTG-3'

Intron7 reverse primer: 5'-TGTTCTCTAGGGCTTCCTCATC-3'

pTARBAC2.1_1 forward primer: 5’- ATAAATCCTGGTGTCCCTGTTG-3’

pTARBAC2.1_1 reverse primer: 5’- TCCTTAGCTCCTGAAAATCTCG-3’

*Transgene genotyping assay*

The final genotyping protocol for the transgene used the Intron7 primers and cycling conditions listed above with following modified protocol. To isolate DNA from mice, tail snips (0.5 cm) were placed in 250uL - 300uL DirectPCR Lysis reagent (Viagen 102-T) with 10uL of 10mg/mL Proteinase K (Invitrogen 25530-015) overnight at 55°-60°C. The crude DNA preps were diluted 1:10 in H_2_0 and then 5 μl was used as templates in the PCR reaction. PCR reaction was carried out in 25uL total volume with a final concentration of 1X reaction buffer, 2mM MgCl2, 0.2mM dNTPs, 0.5 units of Platinum Taq (Invitrogen), and 0.8 μM of each primer. The presence of the transgene was detected by a ~150 bp amplicon that is not present in wild-type mice.

*Hprt1 genotyping assay*

A multiplex PCR assay similar to one described in [1] was used to determine the genotype of the mice at the *Hprt* locus. In this case, the *Hprt1^b-m3^* and wild-type alleles were detected by the presence of a 1.2 and 1.0 kb amplicons, respectively. The PCR cycling conditions were identical to those used for the transgene and the primer sequences are:

WT forward: TCA TGG ACT GAT TAT GGA CAG G

DEL forward: ATC GAT TTG GTA GCA CTG GAA G

WT/DEL reverse: TTA TAC AGC CTC GAT GGA CAA C

*RT-PCR assay used for initial confirmation of the expression of the transgene*

RT-PCR was performed on cDNA generated from DNase treated total RNA from the four transgenic lines using *PRTFDC1*-specific and control primers (*Hprt1*) to confirm the expression of this gene in each transgenic line. Reaction and cycling conditions followed the protocol described for the BAC PCR assays.

Primer sequences:

hsPRT bac_2f (spans exon 4-5 splice junction): GCT GGA AAG AAT GTT CTC ATT G

hsPRT bac_2r (exon 8): GGC ATA TCC CAC CAC AAA TAA G

mmuHPRT1f (exon 7): AAG TGT TGG ATA CAG GCC AGA C

mmuHPRT1r (exon 9): TTA CTG GCA ACA TCA ACA GGA C

**REFERENCES**

1. McEwan C, Melton DW (2003) A simple genotyping assay for the Hprt null allele in mice produced from the HM-1 and E14TG2a mouse embryonic stem cell lines. Transgenic Res 12: 519-520.
