# Supplementary figures and images for: PRTFDC1 Is a Genetic Modifier of HPRT-Deficiency in the Mouse
Source: PLoS One. 2011 Jul 27;6(7):e22381. doi: 10.1371/journal.pone.0022381 (PMC3144895; doi:10.1371/journal.pone.0022381)

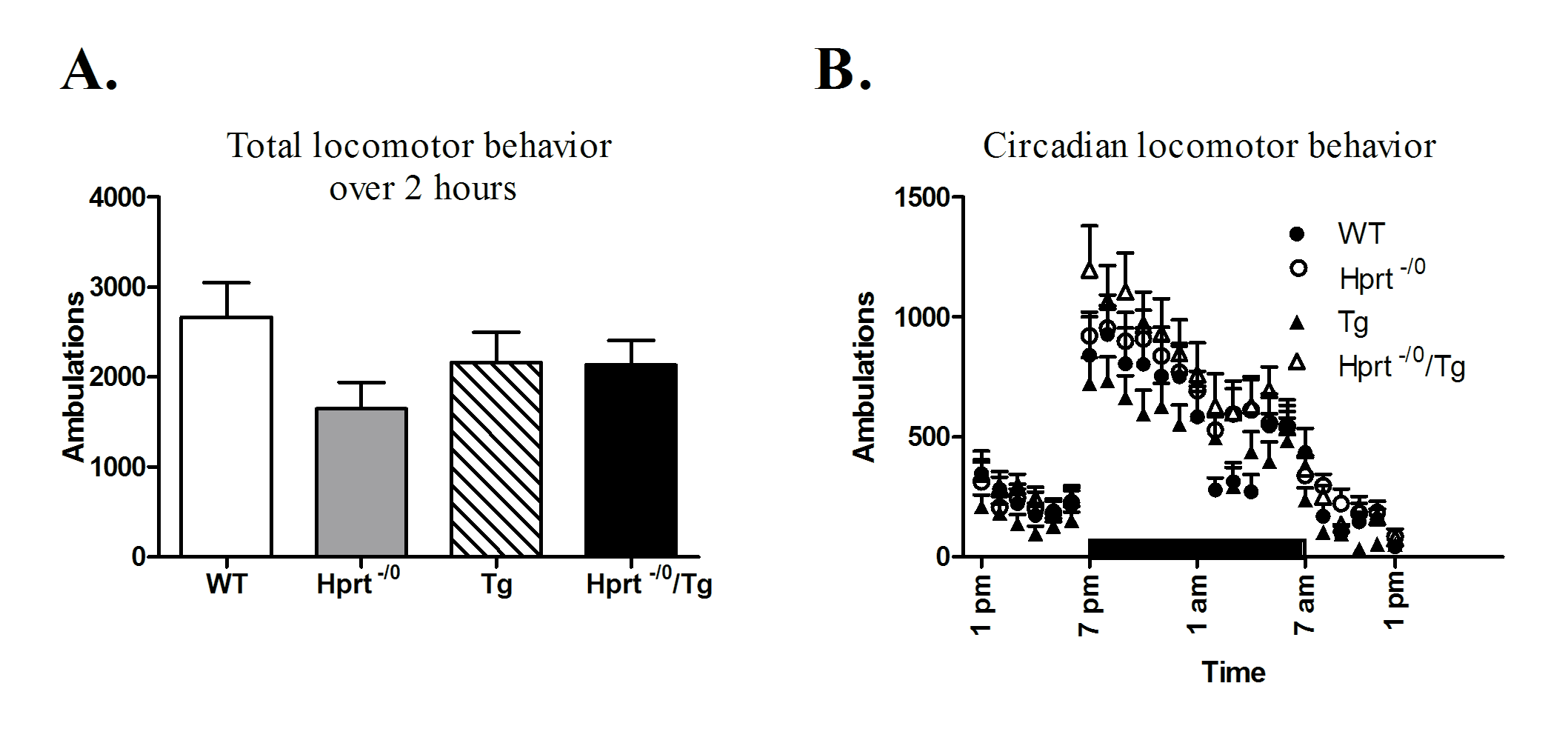

Supplement: Figure S3 — Neither deficiency of HPRT nor the presence of the PRTFDC1 transgene affect locomotor behavior. Mice were placed in locomotor-monitoring chambers and ambulations were recorded for 2 (a) or 24 (b) hours. Shown are mean ± SEM ambulations (consecutive beam breaks). (TIF) [file pone.0022381.s004.tif]

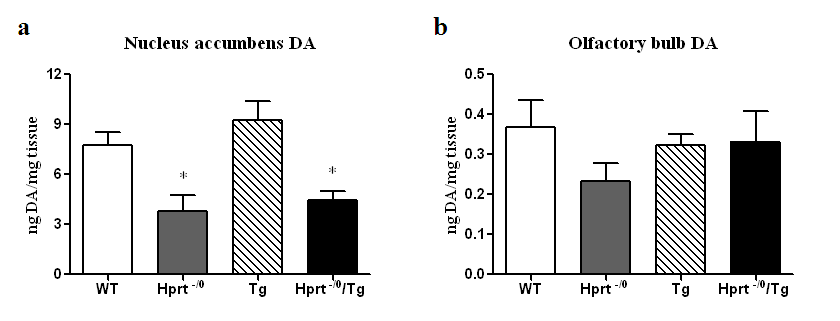

Supplement: Figure S4 — Dopamine levels in the nucleus accumbens and olfactory bulbs. DA levels were measured in males (n = 7–14) from each genotype in the nucleus accumbens (a) and the olfactory bulbs (b). * P<0.05 comparing Hprt−/0 males (with or without the transgene) with wild-type and Tg mice. No significant differences between genotypes were seen in the olfactory bulbs. (TIF) [file pone.0022381.s005.tif]
